# Promoting Data Literacy: Using Social and Emotional Learning Assessment Data to Inform Teaching and Learning

**DOI:** 10.3390/bs15121728

**Published:** 2025-12-13

**Authors:** Zi Jia Ng, Britney Foster, Troya L. Ellis, Sophie P. Barnes, Jessica D. Hoffmann, Christina Cipriano

**Affiliations:** 1 Yale Child Study Center, Yale University School of Medicine, New Haven, CT 06511, USA; sophie.barnes@yale.edu (S.P.B.); jessica.hoffmann@yale.edu (J.D.H.); christina.cipriano@yale.edu (C.C.); 2 Trajectory of Hope, Los Angeles, CA 90056, USA; britney.foster@trajectoryofhope.com (B.F.); troya.ellis@trajectoryofhope.com (T.L.E.)

**Keywords:** social and emotional learning, data literacy, assessment data

## Abstract

Data literacy enables members of a school to evaluate the credibility of the information they encounter, meaningfully interpret the findings in context, and empower stakeholders to make data-based decisions that can promote student success. However, schools are often not equipped to readily interpret, critically appraise, and appropriately use data. This is particularly true for data from social and emotional learning assessments that measure non-academic skills and behaviors crucial to students’ well-being and success. This study illustrates the use of advise-and-design sessions, a sequence of focus groups that unpack, make sense of, and act on assessment data to inform and improve teaching and learning. A total of 37 educators and 11 adolescent students from 12 schools participated. Each was involved in 4–5 advise-and-design sessions during Spring 2024. The sessions were audio-recorded and then analyzed by thematic inductive coding. Key findings included data-driven introductory positioning, in-depth data review, and data-informed actions. An advise-and-design protocol is developed for use in schools. Implications for teaching and learning practices are also discussed.

## 1. Introduction

Data literacy plays a critical role in schoolwide continuous improvement cycles and reflects the ability to understand, analyze, and interpret data effectively to inform decisions and resolve problems (Mandinach & Gummer, 2013). It empowers individuals and organizations (such as educators and schools) to use, transform, and communicate data in an accurate and meaningful manner (Wolff et al., 2016), which generates descriptive (i.e., what happened?), diagnostic (i.e., why did it happen?), and prescriptive (i.e., how can we do something about it?) insights into systems. Educators, however, are often not equipped or supported to readily interpret, critically appraise, and appropriately use data to inform decision-making in schools (Dodman et al., 2023; Kippers et al., 2018). This is particularly true for data from Social and Emotional Learning (SEL) assessments, which are highly heterogeneous in terms of their constructs, factor structures, and scoring, and range widely in their reliability and validity within and across education systems (Cipriano et al., 2023; McKown, 2019). For example, variability in endorsements as positive or negative relative to a given construct (e.g., is the assessment capturing a skill increasing or the presence of a behavior decreasing) confounded in composite scores (e.g., an overall ‘SEL’ score) complicates the understanding and interpretation of SEL data.

Difficulty understanding or acting on SEL assessment data inhibits transformation of raw data into actionable insights that inform teaching and learning (Ng et al., 2022). Arguably, SEL assessments are of little value if the data they produce cannot be translated into clear actionable insights that are meaningful to schools (Cipriano et al., 2020). Data literacy enables educators and students to evaluate the credibility of information they encounter, interpret its meaning in context, and empower data-driven decision-making (Goodman & Ord, 2025). This is especially important in the promotion of social justice by recognizing and embracing diversity in different ways of learning and being that can show up in SEL assessments. This differentiation is of grave importance for SEL in the current socio-political landscape, where SEL is caught up in the politicization of education in the United States (McCarthy et al., 2025; Tan & Wegmann, 2022). The politicization of SEL is shaping stakeholders’ trust and quieting the study of differential effects under the false pretense that the study of difference is inappropriate practice. In the absence of meaningful data that is differentiated and interpretable and mobilizes decision-making in schools, misinformation about SEL can proliferate and result in harmful practices or policies in schools. Building a culture of SEL data literacy among school-based practitioners can drive evidence-based decision-making and meet the moment of sociopolitical divisiveness with meaningful and actional evidence that supports all students to succeed in school, academically, socially, and emotionally.

The goal of this study is to illustrate how data literacy can be promoted in schools through advise-and-design sessions to inform teaching and learning. Advise-and-design sessions are a sequence of focus group discussions that aim to unpack, make sense of, and act on school-based assessment data with educators (including school leaders) and/or students to inform teaching and learning. This study examined how educators and students understand and analyze data from three different SEL assessments (i.e., educator well-being, student emotion regulation, school climate). The focus on SEL assessment data is purposeful given that schools tend to be less familiar with and less equipped to use and communicate data from measures of non-academic skills and behaviors (McKown, 2019).

### 1.1. SEL Assessments in Schools

SEL assessments include formative (progress monitoring) and summative (achievement evaluation) measures that capture a broad range of social and emotional outcomes (Denham, 2015; McKown, 2019). Student-level measures of social and emotional skills and behaviors are the predominant branch of SEL assessments, such as child/adolescent measures of emotion understanding (Voltmer & von Salisch, 2017), self-control (Duckworth et al., 2014), and perspective taking (Hall et al., 2021). Educator-level measures of social and emotional dispositions and capacity are the alternative branch of SEL assessments, such as adult measures of emotional intelligence (Stillman et al., 2018), relationships (Ferreira et al., 2020), and well-being (Fox et al., 2023). School-level measures of social and emotional experiences and instruction are the collective branch of SEL assessments, which include measures of school climate (Wang & Degol, 2016) and SEL pedagogies (Zieher et al., 2024).

SEL assessments vary widely in terms of who completes them and how they are administered, ranging from self-reports and other-reports to observation methods and direct assessments (McKown, 2019). Each methodology carries strengths and drawbacks. For example, emotional intelligence (EI) self-reports are short and simple to administer; however, self-reported EI skills have been shown to have little association with performance on EI ability tests (Brackett et al., 2006). Another essential consideration in SEL assessments is recognizing the multi-level nature of the data collected to provide a comprehensive snapshot that informs decisions and resolves problems related to strategic initiatives and schoolwide practices (Thapa et al., 2013). Student-level SEL data offers granular insights that can identify learning needs and monitor learning growth beyond academics, which informs decisions on SEL implementation. However, individuals may be uncomfortable with their SEL data being shared with their teachers, and the selection of measures demonstrating measurement invariance is limited. Teacher-level SEL data offers detailed information that guides teaching practices and determines teaching support, which informs decisions on SEL resource allocation, though teachers must feel safe to share their genuine experiences without fear of workplace retribution. School-level data offers comprehensive insights into the learning and teaching environment, which informs decisions on SEL initiatives and school policies, but can sometimes hide or dilute group differences when sufficient breakdowns are not provided. Together, the multi-level data from SEL assessments have the potential to promote an inclusive and supportive educational environment, when understood and applied thoughtfully.

### 1.2. SEL Assessment Frameworks in Practice

Positive Behavioral Interventions and Supports (PBIS) is a framework used in schools to create a positive and supportive environment that promotes social, emotional, and behavioral success for all students (Barrett et al., 2018). It is a part of the overarching Multi-Tiered Systems of Support (MTSS) designed to provide comprehensive academic and behavioral support. Within PBIS, schools usually use data from SEL assessments to determine students’ social and emotional strengths and needs, determine focus for schoolwide SEL supports, guide effective teaching and learning practices, and monitor students’ progress from universal SEL interventions (Brann et al., 2022; McKown, 2019). Schools may also use data from SEL assessments to better understand school culture and climate from the perspectives of students, educators, support staff, and administrators to enable a positive and supportive learning and working environment for all.

The systemic SEL model is a unifying preschool through high school framework established by the Collaborative for Academic Social and Emotional Learning (CASEL). It centers on five core social-emotional competencies—self-awareness (e.g., understand one’s thoughts, feelings, and actions), self-management (e.g., regulate one’s thoughts, feelings, and actions to achieve goals), social awareness (e.g., feel and show care and concern for others), relationship skills (e.g., build and maintain healthy relationships), and responsible decision-making (e.g., make constructive choices)—to enhance students’ capacity to integrate cognition, affect, and behavior to adaptively handle daily tasks and challenges within and beyond school (Weissberg et al., 2015). The systemic model involves coordinated classroom, school, family, and community efforts as well as district, state, and federal policies and support to provide quality schoolwide SEL implementation. It also encompasses assessing short-term and long-term attitudinal and behavioral outcomes from evidence-based SEL implementation.

Data from SEL assessments can be a powerful resource for ongoing, proactive efforts to enhance academic success and advance educational equity in schools when it provides trustworthy results and insights beyond academic accomplishment indicators. There is a compelling need to promote data literacy in schools to support selection of high-quality SEL assessments, the ability to evaluate the veracity of results, and the transformation of raw data from SEL assessments into meaningful and actionable information that advance teaching and learning (Ng et al., 2022). In the context of SEL assessments, data literacy can not only facilitate tracking of student development and achievement, but also drive advancement in quality and equity of educational practices (Dodman et al., 2023; Elisa Raffaghelli, 2020; Gueldner et al., 2020). Data literacy is crucial in schools because it empowers educators and students to readily interpret and critically analyze data to make informed decisions.

### 1.3. Data Literacy Frameworks in Education

Based on the inquiry cycle of Gummer and Mandinach’s (2015) data literacy conceptual framework, educators and students need to identify problems and frame questions and then know how to use data, transform data into information, and transform information into decisions. For how to use data, educators and students need to comprehend the intended purpose of the data, quality of the data (accuracy and completeness), and properties of the data. For how to transform data into information, educators and students need to identify patterns in the data, make meaning of the data, and draw inferences from the data. For how to transform information into decisions, educators and students need to determine next steps (plan, modify, or adapt school practices) and understand the context (circumstances and consequences) for a decision. While Gummer and Mandinach’s (2015) data literacy conceptual framework does not directly address educational equity, opportunities exist to embed equity into the data literacy process, particularly in the problem identification and question framing phase. Equity in data use can be understood as using data to promote educational opportunities by minimizing barriers to student well-being and success (Cipriano & Strambler, 2024). Schools may address disparities in student outcomes by making decisions on data that do not reinforce biases or perpetuate inequalities (Dodman et al., 2023). Equity in data use opens doors, especially for students who have been historically marginalized or underrepresented, by identifying gaps in achievement and well-being or access to and provision of necessary support or resources (Datnow et al., 2017).

According to Datnow and Park’s (2018) data use for equity framework, data should be used by schools to identify disparities and devise ways to address educational inequities. They further recommend collaborative efforts where educators, administrators, students, families, and community partners work together to create meaning and guide practice and policy. This framework moves emphasis from a broad generic approach to an equity-centered approach, which positions educators, students, and others as active agents in advancing educational equity through data-driven decision-making processes. Adopting both Gummer and Mandinach’s (2015) data literacy conceptual framework and Datnow and Park’s (2018) data use for equity framework strengthens the conceptual grounding for this study because each offer something essential yet distinct. Gummer and Mandinach (2015) provide a foundational model of the data literacy process and the knowledge and skills required to engage in each step. Datnow and Park (2018) meaningfully extend this model by introducing an equity-centered perspective and orienting data use towards equity and justice. Bringing together these perspectives, this study aims to show how educators and students can use and communicate data from different SEL assessments collaboratively to produce meaningful and actionable insights that inform decisions, resolve problems, and advance educational equity in school.

### 1.4. Purpose of the Study

Data literacy empowers educators and students to evaluate the credibility of information they encounter and make decisions that are based on actively including and listening to different voices in school (Wolff et al., 2016). Students bring a unique perspective to the interpretation and use of data grounded in their experiences, interactions, hopes, and beliefs. Students can ideally participate in all parts of the Gummer and Mandinach (2015) framework above by providing input on the problems to address and engaging in the process of transforming data into decision-making. Data literacy stands to benefit all, but it requires intentional actions to educate, support, and sustain efforts to transform raw data into meaningful and actionable insights for educators and students. This study aims to illustrate how data literacy can be promoted in schools through advise-and-design sessions to inform teaching and learning.

The study is drawn from a three-year (2021–2024) Research and Practice Partnership (RPP) between one research university and two community-based school support organizations, with the shared goal of advancing academic success and educational equity through the implementation of SEL assessments. RPP is a long-term collaboration between researchers and practitioners to address problems of practice and develop evidence-based solutions for improving educational outcomes (Coburn & Penuel, 2016). In the 2021–2022 school year, the RPP recruited full-time SEL specialists for partner schools. In the 2022–2023 school year, the RPP introduced SEL specialists into partner schools who implemented different SEL assessments based on school preferences and needs. In the 2023–2024 school year, the RPP tasked SEL specialists to administer advise-and-design sessions at their school. Educators and students were recruited for advise-and-design sessions using convenience sampling.

The primary objective of this study is to illustrate intentional data literacy in schools by documenting how educators (including school leaders) and students unpack, make sense of, and act on data from SEL assessments through advise-and-design sessions. Adopting Gummer and Mandinach’s (2015) data literacy conceptual framework as well as Datnow and Park’s (2018) data use for equity framework, the study addresses three research questions:

(1) How do educators and adolescent students comprehend SEL assessment data?
(2) How do educators and adolescent students evaluate and transform SEL assessment data into information that identifies disparities in teaching and learning?
(3) How do educators and adolescent students transform SEL information into decisions that advance educational equity?

These questions are of practical importance as misinformation about SEL is caught up in the politicization of education in the United States (McCarthy et al., 2025; Tan & Wegmann, 2022). The secondary objective of this study is to develop a semi-structured data literacy protocol based on study findings, which can be utilized with educators and students to promote effective understanding and use of school-based assessment data in schools. This is of practical significance for schools that are often not equipped or supported to readily interpret and appropriately use data from school-based assessments to inform decision-making on teaching and learning (Dodman et al., 2023; Kippers et al., 2018; McKown, 2019).

## 2. Methods

### 2.1. Participants

Schools. Twelve schools across California (two elementary schools and two middle schools), New Jersey (one high school), and New York (seven high schools) participated in this RPP. For all schools, over 80% of the students identified as a racial/ethnic minority (e.g., Asian, Black/African American, Hispanic/Latine). Over 80% of the students came from low-income families (e.g., eligible for free or reduced-price lunch, experiencing housing insecurity).

Educators. All educators (including administrators) from 11 participating schools (two elementary schools, two middle schools, seven high schools) were invited to take part in advise-and-design sessions during Spring 2024. A total of 37 educators across those schools participated in advise-and-design sessions. There were at least three educators at every session for each school. Prior to advise-and-design sessions, 251 educators from 11 of the participating schools (two elementary schools, two middle schools, seven high schools) took the Educator Well-Being (EWB) survey.

Students. Students from three participating schools were invited to take part in advise-and-design sessions during Spring 2024. A total of 11 adolescent students across those schools participated in advise-and-design sessions. There were at least three students at every session for each school. There were fewer advise-and-design sessions with students than educators because students were not privy of educator SEL data. In other words, there were no opportunities for students to take part in advise-and-design sessions when the EWB survey was the only SEL assessment administered in a school.

Prior to advise-and-design sessions, 1278 students across five participating schools (two middle schools, three high schools) took the School Climate Walkthrough (SCW), while 1225 students from eight participating schools (two elementary schools, one middle school, five high schools) took the Student Emotion Regulation Assessment (SERA). Students took the SCW and/or the SERA based on SEL specialists’ discussion with school leadership on school preferences and needs for SEL assessment.

SEL Specialists. A total of nine SEL specialists across 12 schools facilitated advise-and-design sessions during Spring 2024. SEL specialists are full-time staff hired for this RPP and introduced into schools to understand what it takes to effectively implement SEL assessments and co-learn with decision makers from within the school walls. The key role of SEL specialists is to identify, implement, and evaluate how SEL assessments can be integrated into extant school system, culture, and policy to document and address challenges and opportunities for supporting SEL implementation and advancing social justice. See Table 1 for the positionality statements of the SEL specialists.

### 2.2. Measures

SEL Assessments. Three different SEL assessments were available for administration based on school needs and wants. The Educator Well-Being (EWB) survey is a three-part self-report survey that assess educators’ (a) SEL practices at both individual and classroom levels, (b) affective experiences at work, and (c) sources of well-being at work (Floman et al., 2023, 2024; Strambler et al., 2023a, 2023b). The School Climate Walkthrough (SCW) is an online interactive assessment that captures students’ opinions and observations of their school’s safety, relationship quality, diversity, and teaching quality over the course of a typical school day (Hoffmann et al., 2022). The Student Emotion Regulation Assessment (SERA) is a digital vignette-based assessment that measures students’ use of eight different strategies to manage anger, anxiety, boredom, and sadness across commonly occurring academic and social situations in or related to school (Ng et al., 2025). All three SEL assessments provide aggregated data reports for schools.

Advise-and-Design Sessions. The advise-and-design sessions are a sequence of semi-structured focus group discussions that unpack and make sense of SEL data with students and/or educators (including school leaders) from the same school to inform teaching and learning. Advise-and-design sessions provide a unique platform for students and educators to engage in a reflective process of reviewing, contemplating, and acting on what they learned from SEL assessment data. Each session consists of 4–5 people, which was large enough to gain varied perspectives but small enough to be productive (Gibson, 2012). Adapted from CASEL’s data reflection protocol, main questions included (a) what do you see? (b) what does the data suggest? (c) how may the data inform learning or teaching? and (d) what are the next steps? Additional questions were posed to facilitate the flow of the session and elicit more in-depth responses. Each session took 45 to 60 min and was facilitated by a SEL specialist in person with students and/or educators using SEL assessment data collected at their schools.

### 2.3. Procedures

The study was approved by the institutional review board of the first author’s institution and the institutional review board of New York City Public Schools. It was conducted between February and June 2024. The study included 35 educator sessions across nine schools, 16 student sessions across three schools, and two educator-and-student sessions across two schools. All sessions were conducted after school to ensure no disruption to instructional time. SEL specialists were provided with SEL assessment data reports generated by the research team. They were given flexibility in facilitating the advise-and-design sessions. Apart from the main questions, there was no predetermined format or resources for the sessions. Participants were provided with written and verbal explanations of the nature and purpose of the study, and assured of the confidentiality of data to encourage candor and open sharing. They were assigned a pseudonym to ensure confidentiality. Audio recordings were transcribed with Trint software version 1. Transcripts were analyzed with Dedoose software version 10. Data can be obtained by emailing the last author.

### 2.4. Analysis

Two coders analyzed each field note independently, and a third coder reconciled them. Disagreements were resolved through weekly group discussions, which involved the third coder pointing out the coding discrepancies, the first and second coders sharing their rationale for the code that they gave, and the group coming to a consensus about the final code. Each coder provided a positionality statement to note how their identities and worldviews may have shaped interpretation of the data (see Table 1). Open coding was conducted to identify significant concepts and generate codes, enabling exploration and discovery, as researchers remain open to emerging themes without predetermined concepts (Williams & Moser, 2019). In vivo coding was used to capture participants’ perspectives by using their words verbatim to establish open codes, allowing researchers to maintain the authenticity and richness of participants’ expressions (Williams & Moser, 2019). Codes were refined to add additional details if a coder had difficulty identifying or distinguishing them. Axial coding was then conducted to identify relationships between codes with the use of diagrams or matrices to visualize connections, enabling researchers to establish linkages between concepts and explore how concepts relate (Williams & Moser, 2019). See Table 2 for the codebook. Thematic analysis was utilized to identify and interpret recurring patterns of meaning guided by research questions and the extant literature on data literacy (Braun & Clarke, 2012). Only themes documented for at least half of the schools were reported for brevity and to highlight salience across educators and students.

## 3. Results

Key themes emerged from the introduction of, review of, and action from advise-and-design sessions. The introduction category had four components, with SEL specialists providing (a) a session purpose, (b) an assessment description, (c) community norms, and a (d) community builder, which facilitated educators and adolescent students’ understanding of SEL assessment data. The review category had six themes, with educators and students sharing their perspectives on SEL data in relation to (a) participation rates and demographics, (b) questions about the data, (c) differences across group or time, (d) areas of strength and growth, (e) connection with day-to-day teaching or learning, and (f) potential influences, which enabled educators and adolescent students to transform SEL assessment data into information that identifies disparities in teaching and learning. The action category had three themes, with educators and students sharing their perspectives on next steps with SEL data, including (a) what to improve for SEL practices, and (b) how to improve those SEL practices, which allowed educators and adolescent students to transform SEL information into decisions that advance educational equity.

### 3.1. Educators and Students’ Understanding of Data (RQ1)

Session purpose. SEL specialists described the overarching goal of advise-and-design sessions—to understand, review, contemplate, and act on data from SEL assessments—at the first session. For example, a SEL specialist in School A indicated to a group of educators that “*In these sessions, we’ll go over data from SEL assessments that we’ve taken as a collective of the school, then we’ll strategize ways to implement that data moving forward. That’s going to be our main purpose*.” SEL specialists further stated the specific goal of each advise-and-design session. This often included a quick recap of the previous session. For instance, a SEL specialist in School C mentioned in the second session that “*Last session, we looked at some data. We just tried to get familiar with the data. Today’s goal is to start identifying areas of need*.”

Assessment description. SEL specialists described the assessments administered to students or educators. This often happened at the first session, with context provided for the data that was to be reviewed. For example, a SEL specialist in School B briefly defined the SERA: “*We’re going to talk about the SERA… looks at emotion regulation strategies like avoidance, acceptance, somatic relaxation*.” Another SEL Specialist in School D outlined the EWB, “*It was made up of three different parts. One looked at SEL practices you do in your classroom. The second part looked at SEL practices you do individually with your own self. The last part looked at how you feel in the workplace… and what’s contributing to how you’re feeling*.”

Community norms. SEL specialists established norms during the first session to guide how individuals communicate and interact during advise-and-design sessions. Community norms are shared expectations and standards of behavior, which create and maintain a space that promotes harmony, safety, and belonging. For instance, a SEL specialist in School A co-created norms with the group: “*Let’s establish some kind of community norms. They’re basically unspoken rules of how we’re going to treat each other and how we’re going to show up to this space… Because this is our space*.” SEL specialists also reminded the group of established norms during subsequent sessions. Another SEL specialist in School H noted community norms at the beginning of the third session: “*It’s fine to be hard on the content, but be soft on the people. Focus on learning, not judging… Take the lessons, but leave [personal] stories behind… to remember that people speak in drafts. The first thing that we say may not be everything that we meant to say.*”

Community builder. SEL specialists built rapport and engaged students and educators with a connection activity at each advise-and-design session. This can foster connection and collaboration. For example, a SEL specialist in School C did a community builder related to data literacy: “*Before we dive into the data, let’s do a temperature check on how people feel about data. Sometimes people love data. Some people are a bit skeptical. So maybe you can share a word or phrase that comes to mind when you think of the word ‘data’.*” Another SEL Specialist in School I had a community builder based on SEL, “*Before we get started, I have a Mood Meter here. If you could share how you feel… You can choose a color if you don’t have a specific feeling.*”

### 3.2. Educators and Students’ Transformation of Data into Information (RQ2)

Participation rates and demographics. Educators and students shared thoughts and feelings about sample size and demographics of the data (e.g., gender, race, grade level range, general or special education). Some wondered how sample size and demographics may influence the data as they evaluated how much to trust the results, or weigh certain results when making meaning. For the EWB, an educator in School H mentioned, “*I wonder if this data will look similar in another school or different state. I also wonder how years of experience influence the data*.” Some also noted that the data may not be representative of the school population, indicating skepticism about validity. When reviewing data from the SERA, a student in School A pointed out that the sample mirrored race but not age characteristics of the larger school population: “*So only there’s only two Middle Eastern or North African and one American Indian or Alaskan Native… The largest group is Hispanic or Latin American. To be honest, mostly everyone I see speaks Spanish as a first language*.” It is erroneous to assume that data reflects the cultural heterogeneity of a school. Equity in data calls for adequate and inclusive representation of different groups of students or educators, especially those who have been systematically ignored or excluded (Datnow et al., 2017).

Questions about the data. Educators and students asked questions about the data. For the EWB, an educator in School I indicated, “I found this [data report] a little bit challenging to understand… I had questions about these numbers.” Some struggled to interpret the Likert scale. An educator in School B questioned, “When it says 4.29, what’s it out of? What does it mean?” Likewise, a student in School B asked, “Is three [on a four-point scale] good or bad?” Misinterpretation of data may be attributable to unfamiliarity with SEL indicators, lack of normative SEL assessment data, and misalignment between SEL assessment report’s statistical complexity and students/educators’ statistical literacy. Questions did not come up about the psychometric properties of the assessments, where they were developed, or how they were selected, indicating another potential gap in the participants’ data literacy to fully evaluate the data.

Differences across groups or time. Educators and students shared thoughts and feelings about group differences in data. For the SCW, an educator in School B indicated, “*I think that the glaring thing for me is our marginalized communities. Our African-American students and our non-binary students are not feeling connected or supported*.” Educators and students also discussed differences in data across time. Another educator in School B noted, “*There were some standouts in changes for me. I saw a 10% increase in unkind language in classrooms, and a 15 to 20% increase in students feeling that they were treated unfairly because of who they are*.” Data use for equity involves identifying disparities in academic achievement and opportunity, such as gaps in outcomes or differences in treatment between groups of students and/or educators (Datnow & Park, 2018). This in turn engenders equity-focused change in schools.

Areas of strength and growth. Educators and students reflected on high and low scores in the data. For the SCW, an educator in School D pointed out, “*A glow was that 84% of students said that teachers were making an active effort to build positive relationships with their students during class and between classes…. An area of concern a huge portion of kids feel there are spaces in school where people are not treated fairly. Most often was at the school entrance.*” For the EWB, an educator in School C indicated, “*The biggest thing that I found was the educator-leadership relationship was lower than educator-student or educator-colleague relationships. For example, at least a quarter had a negative response to personal connection to leadership*.”

Connection with teaching or learning. Educators and students shared thoughts and feelings about how the data related (or did not relate) to their experiences. For the EWB, an administrator in School A noted, “*I want to bring attention to educator-family relationships. Educator-student relationships scored high, but not educator-family relationships. We must partner with not just our kids, but also our parents… It’s reflective of our turnout when we have certain workshops or events for parents you know*.” For the SCW, where 54% of students at School J rated that an educator checked in with them outside of grades during the school day, a student in School J expressed that this result did not align with their experience: “*I missed like two weeks of school. Two teachers called to make sure that I wasn’t really behind everybody. So, they were sending me work on Google Classroom and making sure that I was catching up with other classmates. It was only like two teachers, and it was about schoolwork*.” Another student in School J added, “*Personally no teacher would call home because I missed school. I think in total I missed like a week in school. Yeah, no teacher called*.” By comparing the results to their own experiences, students may share their skepticism of the data’s validity, or share their surprise that so many of their classmates responded differently than they did. Making such connections allows deeper understanding of the lived experiences of different groups of students and/or educators (Datnow & Park, 2018). Group differences may be attributable to differential challenges, opportunities, constraints, and support at school.

Influences on the data. Educators and students shared thoughts and feelings about school and individual influences on the data. For the SERA, an educator in School I indicated that data trends may be attributed to SEL programming, “*Problem solving is the strategy that students most frequently use to manage their emotions. And to me, what this is telling me is that they’re learning Second Step. They’re learning ‘What are the things I need to do when there is a problem?*’.” This statement also suggests that the educator understood their school’s data to be a reflection of their specific practices, and not necessarily generalizable to how all students would respond. For the EWB, an administrator in School A noted the importance of adult modeling: “*I recently saw a staff member teaching. She was struggling and she said to her class like, ‘I’m struggling right now. I’m getting really frustrated.’ It was huge… She was modeling how it’s okay to feel frustrated*.”

Educators and students also shared observations about demographic differences in the data; on top of what they had noted about participation rates, they also shared their hypotheses about how observed disparities might be systemic. An administrator from school F commented on the EWB: “I noticed that teachers of 6 to 10 years [of experience] had the smallest percentage of the ones who completed the survey. Maybe they’re overwhelmed. Maybe they’re disconnected. But to me, it was so interesting that the middle ones had the smallest amount of participation.” A student in School A expressed that gender differences in data may be due to gender roles: “Emotional support-seeking for boys is 42.3%. And then for girls it’s like 72.5%… If you really think about it, most girls are usually in groups, and they have things they relate to, but boys I don’t know. They kind of like, ‘Hey, do you want to play soccer? Let’s go.’ We [boys] just don’t have that many friends… Guys are built up to be tough.”

### 3.3. Educators and Students’ Transformation of Information into Decisions (RQ3)

What to improve. Educators and students shared their perspectives on areas for improvement in their schools based on the data. For example, educators in School G identified several areas of growth for students: “*Too many students are challenge-averse… Too many students are unable to identify or regulate their emotions… Too many students are not connecting their learning now to their future*.” Similarly, educators in School C recognized different areas of growth for administrators: “*For admin-faculty dynamics, one of the things that I hear a lot about is there’s not always follow through with things. Like, things will be brought up to the admin, but then it doesn’t get moved along… I feel out of the loop of decisions that are made and conversations that are happening…*” Students in School B targeted one key area for action planning, “*It’s important for passing periods to be longer… would provide us with more time to move between classes, check in with teachers and friends [emotional support-seeking], and take necessary breaks [somatic relaxation]… which then reduce tardiness and stress.*”

How to improve. Educators and students shared their perspectives on how to improve target areas based on effort and impact. For instance, educators in School G suggested that disengagement in school may be due to students’ disconnection between learning and future: “*Because of circumstances our students don’t always have the same opportunities to imagine themselves in the future like other students do. We need to be more intentional… being a lot more explicit about how things in school can possibly help them in the future. So, thinking about if there’s a place in our lesson plan where we could say,* “*We’re learning this literacy strategy today because if you ever need to read a contract in the future. So, making those connections…*” With their need for more time for emotion regulation, students in School B proposed, “*We should change passing periods to 7 minutes… expand the school day [by] 15 minutes to have those 7-minute passing periods… Most students often struggle with coming to school earlier, so maybe we add time at the end of the day…*”

## 4. Discussion

In this RPP, SEL specialists facilitated a structured advise-and-design model that supported students and educators in making meaning of SEL assessment data and translating insights into action. Across schools, participants demonstrated growing capacity to move through Gummer and Mandinach’s (2015) data literacy cycle, interrogating the data, interpreting patterns, and proposing contextually relevant responses. The structure of the sessions enabled participants to situate findings within their lived experiences, compare results across student and educator measures, and consider both individual- and school-level contributors to the patterns that emerged.

Although educators and students engaged actively with the data, equity-related insights surfaced inconsistently. Consistent with Datnow and Park’s (2018) framework, equity considerations often required explicit facilitation to emerge, particularly when interpreting disaggregated belonging and climate data. Some educators noted concerns about representativeness, while others articulated disparities they had previously observed but had not felt positioned to raise. The advise-and-design model thus functioned not only as a mechanism for building data literacy but also as a scaffold that legitimized equity-focused inquiry within school teams. In two of the schools, the concluding advise-and-design session convened students and educators jointly, allowing them to interpret findings together and identify feasible adjustments to school routines, such as modifying passing periods or integrating emotion-regulation practices into grade-level activities.

Taken together, these findings suggest that the advise-and-design structure provides a practical and replicable pathway for strengthening schoolwide SEL data use. By positioning students and educators as co-interpreters of data, the model supports more transparent decision-making, enhances trust, and helps generate actionable, context-specific improvements that extend beyond compliance-driven or leadership-only data conversations.

### 4.1. Practical Implications of Findings

Schools might use such a protocol to increase collaborative decision-making by integrating student and educator voices. Increased data transparency can also build trust and increase engagement and valid responding on future assessments administered.

Study findings have significant practical implications for students and educators. A central implication is the value of providing structured opportunities for students and educators to move from data to meaning to action. Reviewing SEL assessment data surfaced insights that were not previously visible to practitioners, including discrepancies between educators’ perceptions of their practices and students’ reported experiences. Intentional facilitation supported participants in interpreting these findings collaboratively and connecting them to concrete improvements in relationships, classroom climate, and instructional routines. This aligns with research demonstrating that structured opportunities for data use strengthen educators’ capacity to engage in inquiry and make informed decisions (Gummer & Mandinach, 2015).

Findings also highlight the importance of facilitation. Educators noted that having an external facilitator guide data conversations reduced pressure on school staff and created a neutral, supportive space for inquiry. This finding corresponds with prior work emphasizing that effective data use requires collaboration, psychological safety, and structures that make data interpretation accessible to diverse stakeholders (Datnow & Park, 2018). While equity considerations did not consistently surface without explicit prompting, the facilitated structure occasionally opened space for recognizing disparities in experiences across student groups—an observation that reflects broader challenges in integrating equity into routine data-use practices noted in SEL research (Zieher et al., 2024; Cipriano et al., 2025).

Based on these findings, an advise-and-design protocol (see Table 3) can support schools in integrating student and educator voices into continuous improvement efforts. Schools might use such a protocol to strengthen collaborative decision-making, build trust through transparent data-sharing, and increase valid responding on future assessments. By intentionally structuring data discussions and providing facilitation, schools can create the conditions for meaningful, shared interpretation of SEL assessment data and more equitable, responsive schoolwide practices.

Second, data-driven conversations highlight areas for action and improvement, ranging from broad goals such as enhancing school-family partnership to specific changes like adding more transition time between classes. It enables prioritization of school initiatives and practices, and deliberation of school policies and approaches. For example, educators across schools would benefit from SEL programming that enhances their capacity to support both their own and their students’ emotional well-being. Importantly, creating spaces for data-driven conversations elevates student and educator voice and agency in making school environments supportive and inclusive for everyone. It allows students and educators to have their opinions, ideas, and needs heard, and engage in open and meaningful dialogs about school. Furthermore, schools might use their data to determine priorities: for example, picking one area for growth as the focus of their efforts for that academic year, or choosing to redouble their investment in an area of strength their community already possesses.

Third, student and educator participation in advise-and-design sessions demonstrates that all stakeholders can not only meaningfully engage with and interpret data, but also actively generate constructive and actionable insights that meet the needs and wants of the school community. For instance, students and educators were curious about the representativeness and demographics of the sample relative to the school population, and how it might impact data interpretation or inform future data collection. They further noted the significance of underlying contextual factors in interpreting data accurately, although they did not raise questions about the psychometrics of the assessment. This indicates that the presentation and review of school-based assessment data, along with resources and support for data literacy, should evolve over time to reflect the questions, interests, and capacities of stakeholders. It also calls for more data transparency and literacy with both students and educators in schools.

### 4.2. Limitations and Future Directions

While this illustrative case study provides useful insights into student and educator data literacy, and demonstrates how SEL assessment data is relevant to school improvement, it is important to note several limitations. As a case study, the findings are reporting on a small number of schools who were committed to social and emotional well-being as foundational to student success. Thus, study findings may not generalize to other school contexts. For example, schools in which the leaders are not open to critical feedback might decline to use SEL assessments or would not create space for open discussion of SEL data. Alternatively, high-achieving schools with a focus in STEM may have students and educators with higher levels of data literacy and require fewer supports for data interpretation and use than other schools. Moreover, depending on the results of the assessment, holding transparent conversations may be more or less difficult; for example, a school in which the educator well-being (EWB) assessment indicates that trust in the leader is their biggest source of stress will be more challenging for the leader to navigate. In such cases, having a protocol to follow may provide additional structure that is helpful, or a protocol that requires data transparency and open conversation with varying stakeholders may be too aspirational. Future work might seek to replicate study findings with a broader range of schools where there are larger variations in data literacy, educational policies, leadership styles, and openness to SEL, such as rural schools, schools in central and southern regions of the United States, and private schools. In addition, feedback could be gathered on the helpfulness and relevancy of the advise-and-design protocol developed based on study findings (see Table 3).

A further limitation concerns the reliance on SEL specialists’ observational field notes and recorded advise-and-design sessions as primary data sources. Although these sources provided rich insight into how educators and students made meaning of SEL assessment data, they also reflect the positionality of the specialists who facilitated and documented the sessions. Facilitator identity, professional background, and relational dynamics may have influenced which themes surfaced, especially regarding equity-focused discussions that required explicit prompting to emerge. While demographic information supported disaggregated analyses, the study did not include systematic written educator reflections, written student reflections, or independent student and educator interviews outside the sessions, limiting the range of stakeholder perspectives represented. Future research should purposefully incorporate multiple data sources, including written reflections, one-on-one student and educator interviews, or follow-up surveys, to triangulate interpretations and more fully capture how diverse stakeholders engage with SEL data.

Also worthy of further consideration is the limited number of students and educators who participated in the advise-and-design sessions. As demographic, background, and academic information was not collected on them specifically, it is unknown how representative participants were of the school community. Furthermore, participant selection was based on convenience sampling. The issues picked as most concerning, and the solutions generated, will be informed by the experiences of those in the advise-and-design sessions. For example, if the student participants were high-achieving student leaders for whom school is already working well, it might explain their astonishment at the large percentage of students who reported being treated unfairly at school. Similarly, if the educator participants were highly engaged teachers or teachers invested in SEL, it might explain why they were bewildered by the small percentage of colleagues who reported engaging in emotion regulation practices in their classroom. In the future, schools might purposefully choose to gather a representative set of students, including grades, race and ethnicity, gender, and academic engagement and achievement.

Through funding from the RPP, each participating school had a full-time SEL specialist placed on staff. The SEL specialists were not only supported by the research team on how to scaffold and facilitate the advise-and-design sessions, but also present as part of the school community building rapport with students, educators, and leadership for two academic years. This raises the question of whether a full-time SEL specialist is necessary to achieve study findings. Future work should explore how well the semi-structured advise-and-design protocol developed based on study findings can work on its own. Future work should also explore other cost-effective data literacy resources (e.g., instructional videos, asynchronous learning modules, AI-assisted data interpretation) that might be employed to a wide range of schools. It is likely that there is not a single “best practice” for making meaning and taking action on SEL assessment data that will apply across all schools, but rather several pathways to success (digital resources versus in-person meetings; levels of data transparency).

Finally, while the process of understanding and using SEL assessment data to promote a positive learning and teaching environment was examined in this study, it was not focused on potential outcomes. The SEL assessments used in this study were all relatively new, and this project represented one of the first tests of using the data they provide to inform school policies and procedures. As people’s perception of their well-being or sense of safety can change quickly, SEL assessments such as the ones used in this study should be treated as a snapshot in time. Future studies should track the assessments’ stability over time and predictive value, as well as the aftereffects of advise-and-design sessions, including proximal assessments of participants’ satisfaction with the processes of and action plans from advise-and-design sessions, longitudinal assessments of schools’ implementation of action steps, and repeated administration of original SEL assessments (i.e., EWB, SCW, SERA) for desired outcomes.

## 5. Conclusions

Data science is a rapidly growing field, and school leadership is using data more and more to drive decision-making and track progress. When school-based assessment data is transparent, students and educators often have new insights and creative ideas for solving problems and enhancing the school environment. For this to work, however, we need more resources and guidance that support data literacy. This study demonstrates how students, educators, and leaders can come together to understand and use relevant data to inform teaching and learning and to promote a positive educational environment. Beginning with SEL assessment data is a particularly suitable pathway, as everyone has a stake in helping schools be safe and supportive environments where each student and educator can achieve their full potential.

## Figures and Tables

**Table 1:** Positionality statements of SEL specialists.

SEL Specialist 1 identifies as a white woman born and raised in England with a master’s degree in developmental psychology. She has a strong interest in special education, working with children with developmental challenges and advocating for inclusion.
SEL Specialist 2 identifies as an Indian/Pakistani woman who grew up in New Jersey. She holds a master’s degree in educational leadership, and has experience teaching math in high school.
SEL Specialist 3 identifies as a Hispanic man who grew up in a middle-class family in urban New York. He holds a bachelor’s degree in cognitive neuroscience and has experience as a mental health counselor in a community crisis stabilization unit.
SEL Specialist 4 identifies as a daughter of Haitian immigrants who grew up in a Caribbean enclave in New York. She holds a master’s degree in education.
SEL Specialist 5 identifies as a Black woman who grew up with her pastoral father in Louisiana after the loss of her mother at a young age. She holds a master’s degree in social work and is a licensed social worker.
SEL Specialist 6 identifies as a White/Chinese introverted woman who grew up in a middle-class family in Canada. She holds a master’s degree in education.
SEL Specialist 7 identifies as a Hispanic woman who grew up in New Jersey. She is pursuing a master’s degree in business administration, and has experience teaching math in high school.
SEL Specialist 8 identifies as a white woman who grew up in a middle-class family in the suburbs of New York. She holds a master’s degree in social work and is a licensed social worker.
SEL Specialist 9 identifies as a Black cisgender woman who grew up in the suburbs of California. She holds a bachelor’s degree in women, gender, and sexuality studies, and has a teaching credential.

**Table 2:** Codebook.

Codebook	
1. Session Introduction	
1a. Session Purpose	Efforts or actions by SEL Specialists to describe the purpose and goal of advise-and-design sessions to educators or students.
1b. Group norms	Efforts or actions by SEL Specialists to establish group norms—informal or explicit rules that guide how members communicate, interact, and behave.
1c. Connection Activity	Efforts or actions by SEL Specialists to build rapport or encourage interactions with educators or students during advise-and-design sessions.
1d. Assessment Description	Efforts or actions by SEL Specialists to describe SEL assessment that was used to educators or students during advise-and-design sessions.
2. Data Review	
2a. Review Participants of Assessment Data	Educators or students share thoughts or feelings about participant size or demographics for SEL assessment.
2b. Review Group Differences in Assessment Data	Educators or students share thoughts or feelings about group (e.g., gender, race, grade level) differences in SEL assessment data.
2c. Identify Areas of Strengths and Growth in Assessment Data	Educators or students share thoughts or feelings about areas of strength (best scores) and growth (worst scores) based on SEL assessment data.
2d. Connection of Assessment Data to Day-to-day Schooling	Educators or students share thoughts or feelings about how SEL assessment data relates (or does NOT relate) to their schooling experiences and/or school environment.
2e. Context or Mechanism Underlying Assessment Data	Educators or students share thoughts or wonderings about context (e.g., date, time) or mechanism (e.g., SEL intervention or practice) that may underlie SEL assessment data.
3. Action Steps	
3a. Participant Recruitment for Next Administration	Educators or students share thoughts or feelings about sample size, sample demographics, or logistics for the next SEL assessment administration.
3b. What to Target for SEL Efforts	Educators or students share thoughts or feelings about what SEL knowledge and skills to foster based on SEL assessment data.
3c. How to Advance SEL Efforts	Educators or students create an action plan on how to foster SEL knowledge and skills based on thoughts and feelings shared prior about SEL assessment data.

**Table 3:** Advise-and-design protocol.

**Facilitator**: To uphold norms and guide discussions using the protocol **Notetaker**: To capture notes from discussions and provide a recap for the next session
**Session 1:** Data Exploration **Objective:** Introduce A&D session (60 min) **Process:** Provide a purpose and overarching goal for advice-and-design sessions (5 min) ○ Purpose: To unpack and make sense of SEL data ○ Goal: To inform teaching and learning Facilitate a community builder to build collective capacity and engagement (15 min) Co-create community norms to have respectful and productive dialog (15 min) Describe the SEL assessment that was administered (5 min) ○ Provide information on the intended use (“what”) of the assessment ○ Provide rationale for the selection (“why”) of the assessment ○ Provide context on the administration (“how”) of the assessment Have participants explore data to get oriented (20 min) ○ Do they understand what is presented?
**Session 2:** Data Description **Objective:** Dive into SEL data (60 min) **Process:** Recap community norms and previous session (5 min) Have participants review data and take notes independently (15 min) Have each participant share (may pass or concur): ○ One bright spot they observed (10 min) ○ One dark spot they observed (10 min) ○ One thing they noticed about sample size or demographics (10 min) ○ One thing they noticed about group (age, gender, race) differences (10 min) Compile share out on chart paper or anywhere that is visible to everybody
**Session 3:** Data Interpretation **Objective:** Bridge SEL data with teaching and learning (60 min) **Process:** Recap community norms and previous session (5 min) Have participants reflect and take notes independently about what the data suggests about teaching and learning (10 min) Have each participant share (may pass or concur): ○ One possible explanation for a bright spot identified (15 min) ○ One possible explanation for a dark spot identified (15 min) ○ One possible explanation for a group difference identified (15 min) Compile share out on chart paper or anywhere that is visible to everybody
**Session 4:** Data Implication **Objective:** Create action plan to advance SEL efforts (60 min) **Process:** Recap community norms and previous session (5 min) Have each person share one thing they might do next (30 min): ○ One area of SEL knowledge/skill to target ○ One way to improve that area of SEL knowledge/skill Compile the share-out on chart paper or anywhere that is visible to everybody Evaluate ideas for change to determine action plan (25 min) ○ Discuss which ideas are high-impact and low-effort to get a move on ○ Determine which ideas are high-impact and high-effort to work toward

## Data Availability

The data presented in this study is available on request from the corresponding author.
